# BALR-6 regulates cell growth and cell survival in B-lymphoblastic leukemia

**DOI:** 10.1186/s12943-015-0485-z

**Published:** 2015-12-22

**Authors:** Norma I. Rodríguez-Malavé, Thilini R. Fernando, Parth C. Patel, Jorge R. Contreras, Jayanth Kumar Palanichamy, Tiffany M. Tran, Jaime Anguiano, Michael J. Davoren, Michael O. Alberti, Kimanh T. Pioli, Salemiz Sandoval, Gay M. Crooks, Dinesh S. Rao

**Affiliations:** Department of Pathology and Laboratory Medicine, UCLA, Los Angeles, USA; Cellular and Molecular Pathology Ph.D. Program, UCLA, Los Angeles, USA; Jonsson Comprehensive Cancer Center, UCLA, Los Angeles, USA; Broad Stem Cell Research Center, UCLA, 650 Charles E. Young Drive, Factor 12-272, Los Angeles, CA 90095 USA; Department of Environmental Health Sciences, UCLA, Los Angeles, USA; Molecular Toxicology Interdepartmental Ph.D. Program, UCLA, Los Angeles, USA; All India Institute of Medical Sciences (AIIMS), New Delhi, India

**Keywords:** lncRNA, B-ALL, MLL, SP1, Microarray, Leukemia, RNA, Non-coding RNA

## Abstract

**Background:**

A new class of non-coding RNAs, known as long non-coding RNAs (lncRNAs), has been recently described. These lncRNAs are implicated to play pivotal roles in various molecular processes, including development and oncogenesis. Gene expression profiling of human B-ALL samples showed differential lncRNA expression in samples with particular cytogenetic abnormalities. One of the most promising lncRNAs identified, designated B-ALL associated long RNA-6 (BALR-6), had the highest expression in patient samples carrying the MLL rearrangement, and is the focus of this study.

**Results:**

Here, we performed a series of experiments to define the function of BALR-6, including several novel splice forms that we identified. Functionally, siRNA-mediated knockdown of BALR-6 in human B-ALL cell lines caused reduced cell proliferation and increased cell death. Conversely, overexpression of BALR-6 isoforms in both human and mouse cell lines caused increased proliferation and decreased apoptosis. Overexpression of BALR-6 in murine bone marrow transplantation experiments caused a significant increase in early hematopoietic progenitor populations, suggesting that its dysregulation may cause developmental changes. Notably, the knockdown of BALR-6 resulted in global dysregulation of gene expression. The gene set was enriched for leukemia-associated genes, as well as for the transcriptome regulated by Specificity Protein 1 (SP1). We confirmed changes in the expression of SP1, as well as its known interactor and downstream target CREB1. Luciferase reporter assays demonstrated an enhancement of SP1-mediated transcription in the presence of BALR-6. These data provide a putative mechanism for regulation by BALR-6 in B-ALL.

**Conclusions:**

Our findings support a role for the novel lncRNA BALR-6 in promoting cell survival in B-ALL. Furthermore, this lncRNA influences gene expression in B-ALL in a manner consistent with a function in transcriptional regulation. Specifically, our findings suggest that BALR-6 expression regulates the transcriptome downstream of SP1, and that this may underlie the function of BALR-6 in B-ALL.

**Electronic supplementary material:**

The online version of this article (doi:10.1186/s12943-015-0485-z) contains supplementary material, which is available to authorized users.

## Background

The human genome produces thousands of non-coding transcripts [1]. These include the recently described class of long non-coding RNAs (lncRNAs), which have distinct chromatin signatures and epigenetic marks, designating them as unique structures that are conserved in mammals [2, 3]. More recently, comparison of lncRNA expression in zebrafish to that of mammals has suggested that although these structures retain limited overall sequence conservation among vertebrates, they show strong conservation of short stretches of sequence, chromosomal synteny and functional conservation [4]. Prior studies have shown that lncRNAs play a variety of roles in the regulation of transcription, splicing and miRNA function [5–7]. This may not be an exhaustive description of the functions of lncRNAs, as new functions are being discovered in other cellular processes [8, 9]. As might be expected, considering their roles in critical cellular functions, lncRNAs have been found to be dysregulated in cancer, with functional roles in oncogenesis described for a handful of lncRNAs so far [10–13].

B-lymphoblastic leukemia (B-acute lymphoblastic leukemia, B-ALL) is a malignancy of precursor B-cells harboring mutations and translocations that result in dysregulated gene expression [14, 15]. We have recently completed a comprehensive description of lncRNAs in B-ALL and analyzed the association of lncRNA expression with clinicopathologic parameters. Our study showed differential lncRNA expression in samples with particular cytogenetic abnormalities [16]. One of the lncRNAs from our study, designated B-ALL associated long RNA-6 (BALR-6), was significantly upregulated in all subsets of patient samples when compared to normal CD19+ cells. Interestingly, the highest expression of BALR-6 was seen in patient samples carrying the MLL rearrangement [16]. MLL rearranged B-ALL cases have a very poor prognosis and occur in infants, making them particularly hard to treat [17].

**Fig. 1:**
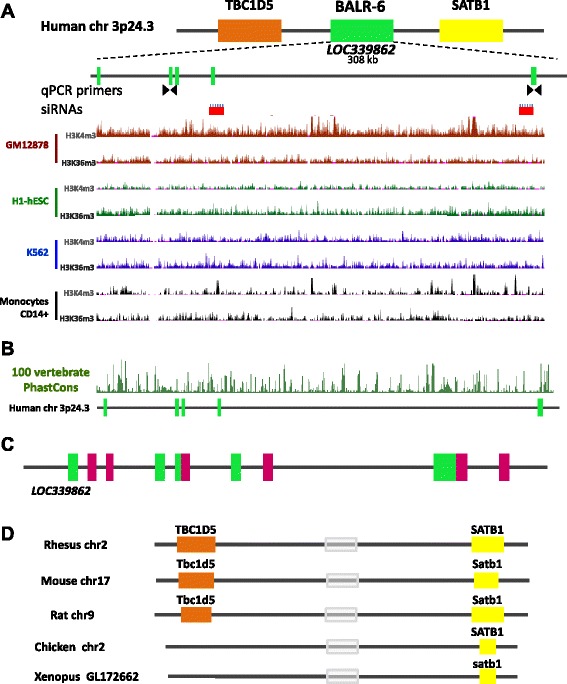
Molecular characterization of *BALR-6*. **a** Top: Chromosomal location of *BALR-6* in the human genome, surrounding genes, qPCR primers, siRNAs, known annotated exons (*green boxes*), known introns (*black lines*) are shown. Bottom: Chip-Seq histone modification map from the Broad institute/ENCODE, taken from UCSC genome browser, shows H3K4m3 and H3K36m3 patterns at *LOC339862* in four different cell types indicating active transcription of the lncRNA. **b** The 100 Vertebrate PhastCons plot from the UCSC whole-genome shows conserved regions among 98 vertebrates including mice and zebrafish throughout the locus. **c** RACE discovered unannotated exons (*magenta*) depicted with known annotated exons (*green*) at *LOC339862*. **d** Schematic depicting genomic conservation of the syntenic block among multiple vertebrates, as analyzed by BLAT. Grey box indicates location of homology to *BALR-6*

Located on chromosome 3p24.3 in humans, *BALR-6* exists in a syntenic gene block with neighboring genes *SATB1* and *TBC1D5* that is conserved in several vertebrate species (Fig. 1a, b and d) [16]. Analysis of publically available data from the Broad Institute/ENCODE shows H3K4m3 and H3K36m3 modifications along the promoter and gene body at *LOC339862*, where BALR-6 resides, indicating that it is a transcriptional element (Fig. 1a) [4, 16, 18–20]. Alternative splicing analysis by the Swiss Institute of Bioinformatics predicted multiple transcripts expressed at this gene locus (Additional file 1: Figure S1A) [21]. Moreover, 100 Vertebrate PhastCons analyses of the *BALR-6* locus demonstrated significant conservation of the gene body, suggesting a functional transcript (Fig. 1b) [22].

To further study this lncRNA we undertook loss-of-function analyses in B-ALL cell lines and gain-of-function analyses in vivo. We found that BALR-6 is a pro-survival factor for B-ALL cell lines, and that its knockdown led to decreased growth and increased apoptosis of these cells. In vivo, overexpression of BALR-6 led to an alteration of hematopoiesis with a shift to more immature progenitor populations. Gene expression analyses of knockdown cell lines showed a differentially expressed gene set in BALR-6 knockdown cells, with enrichment for SP1 transcriptional targets and leukemogenic genes. Finally, luciferase assays demonstrated an increase in transcriptional activity when SP1 and BALR-6 were co-expressed. Together, these findings point to a role for BALR-6 in cellular survival, leukemogenesis and highlight the role of novel elements of gene regulation in B-ALL.

## Results

### BALR-6 knockdown inhibits proliferation of human B-ALL cell lines

To comprehensively study the function for this novel lncRNA, we first characterized the transcripts originating at the genomic locus corresponding to *BALR-6*. Using RS4;11 cell line mRNA, Rapid Amplification of cDNA Ends (RACE) uncovered multiple isoforms; from these, three were cloned and sequenced corresponding to the genomic locus as shown (Additional file 1: Figure S1A-B). Northern Blot analysis of RS4;11 DNAse treated RNA revealed the expression of two isoforms containing exon 3 and exon 5 sequences, one sized at ~3.8 Kb and the other at ~1.2 Kb (Additional file 1: Figure S1C). The annotated mRNA and new alternative splice forms, including unannotated exons, were confirmed as depicted in Fig. 1c. Isoform 1 contains several small open reading frames (ORFs), however no Kozak sequences are found in their initial ATG region, and the predicted ORFs do not resemble any known functional proteins or peptide [23]. Isoforms 2 and 3 lacked open reading frames and translation initiation sites as evaluated by EMBOSS Transeq, predicting them to be non-coding transcripts (Additional file 1: Figure S1D).

**Fig. 2:**
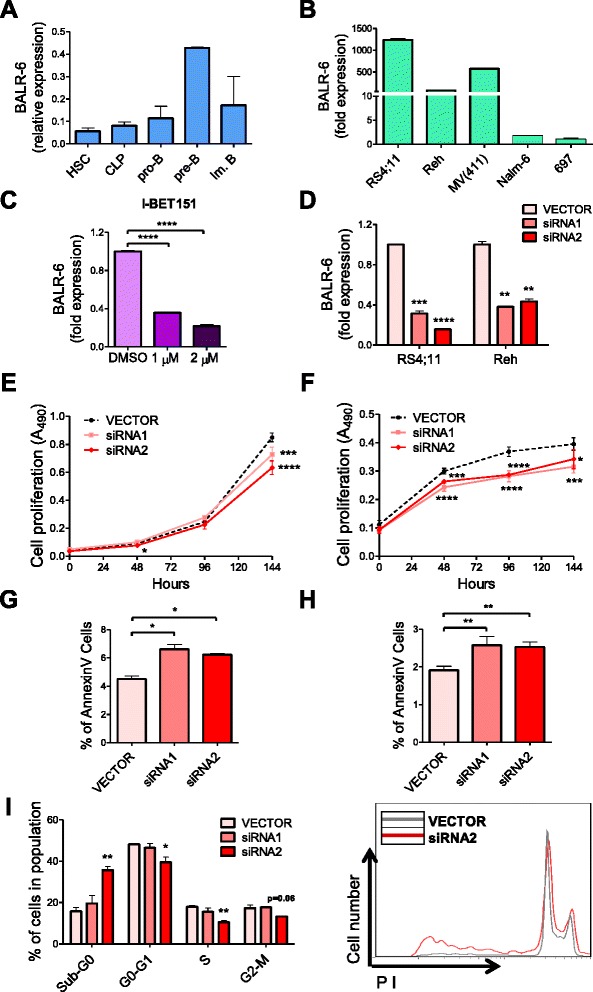
BALR-6 knockdown reduces cell proliferation and increases apoptosis in human B-ALL cells. **a** BALR-6 expression in human bone marrow B-cell subsets by qRT-PCR. Normalized to ACTIN. **b** Quantitation of BALR-6 expression in human B-ALL cell lines by qRT-PCR confirming elevated levels in MLL translocated cell lines RS4;11, and MV(411). Normalized to ACTIN. **c** RS4;11 cell lines treated with 1 μM, and 2 μM of I-BET151 inhibitor for 36 h, presented a decrease in BALR-6 expression levels. Normalized to ACTIN. **d** qRT-PCR quantification of BALR-6 in RS4;11 and Reh cell lines transduced with vector control, siRNA1 or siRNA2. Normalized to ACTIN. **e**, **f** Decreased cell proliferation, upon siRNA mediated knockdown of BALR-6 in RS4;11 cells **e**, and Reh cells **f** as measured by MTS. **g**, **h** AnnexinV staining showed that siRNA mediated knockdown of BALR-6 in RS4;11 cells **g**, and Reh cells **h** resulted in an increase of apoptosis. **i** Propidium iodide staining of RS4;11 knockdown cell lines showed an increase in Sub-G0 and a decrease in G0-G1, S and G2-M cells. Representative histogram of (**i)** confirms cell cycle changes by siRNA2, shown to the right. HSC, hematopoietic stem cell; CLP, common lymphoid progenitor; pro-B, progenitor B; pre-B, precursor B; DMSO, dimethyl sulfoxide. Evaluations were made using a two-tailed *T*-test, *p* < 0.05 (*); *p* < 0.005 (**); *p* < 0.0005 (***); *p* < 0.0001 (****)

To map the murine homologous transcript, we carried out 5′RACE and 3′RACE using mRNA extracted from murine pre B-ALL cell line 70Z/3. The sequences uncovered matched the human BALR-6 sequence, confirming that there is a murine transcript originating from this same locus (Additional file 1: Figure S1E). Further analysis by BLAT showed genomic conservation of syntenic blocks in a variety of vertebrates, including *Xenopus tropicalis* (Fig. 1d). Together, these data demonstrate a highly conserved, functional and complex gene locus that expresses multiple non-coding transcripts, some yet to be discovered. During normal B cell development, BALR-6 is dynamically expressed, with high expression in pre-B cells and subsequent downregulation (Fig. 2a). This suggests that the high expression of BALR-6 in B-ALL could represent a stage-specific expression pattern in leukemia derived from early stages of B-cell development. To elucidate a cellular function for BALR-6, we first evaluated the expression levels of the transcripts in human B-ALL cell lines. BALR-6 expression was highest in RS4;11 cells and MV(411) cells, which carry the MLL-AF4 rearrangement, when compared to other lines (Fig. 2b). Additionally, RS4;11 cells treated with bromodomain and extra-terminal (BET) motif binding protein inhibitor I-BET151 [24] showed decreased levels of BALR-6 in a dose-dependent manner (Fig. 2c). Given that I-BET151 has previously been shown to inhibit transcription downstream of MLL, we propose that BALR-6 expression is induced by MLL, although this effect may not be entirely specific to MLL-AF4.

Using the approach described previously, siRNAs against the splice junctions between exons of BALR-6 were cloned into a mmu-miR-155 expression cassette (Additional file 1: Figure S2A) [4, 16, 25, 26]. We observed knockdown of all the identified transcripts in multiple B-ALL cell lines (Fig. 2d and Additional file 1: Figure S2B). Transduced B-ALL cells showed a reduction in proliferation as early as 48 h after plating, with consistent reduction in proliferation observed over the full duration of the assay (up to 144 h) (Fig. 2e, f and Additional file 1: Figure S2C). siRNA-transduced B-ALL cells had significantly higher levels of apoptosis, as measured by AnnexinV, when compared with vector-transduced lines (Fig. 2g, h and Additional file 1: Figure S2D). Flow cytometry demonstrated that the siRNA2-transduced RS4;11 cell lines had an increase in Sub-G0 cells and a decrease in all other cell stages, consistent with increased apoptosis and decreased flux through the cell cycle (Fig. 2i). Together, these findings suggest a modest yet conserved role for BALR-6 in the regulation of B-ALL cell survival and proliferation.

### Constitutively expressed BALR-6 supports cell survival and proliferation

**Fig. 3:**
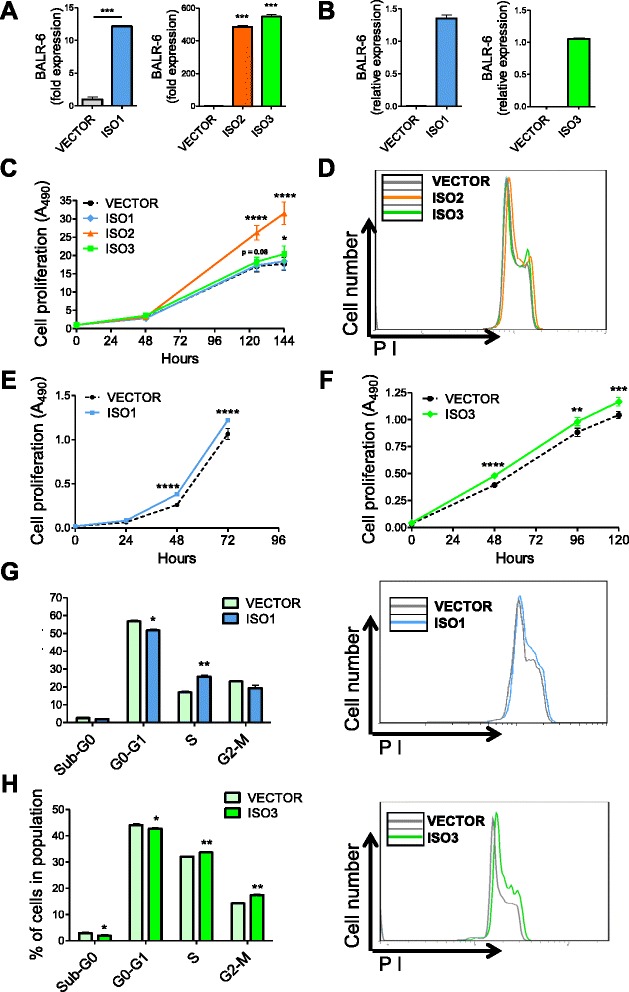
BALR-6 overexpression increases proliferation in human Nalm-6 and murine 70Z/3 cells. **a** qRT-PCR quantitation of BALR-6 isoform expression in Nalm-6 human pre B-ALL cell line. Normalized to ACTIN. **b** qRT-PCR quantification of BALR-6 isoforms in 70Z/3 mouse pre B-ALL cell line. Normalized to Actin (ISO1) or L32 (ISO3). **c** Increased cell proliferation in BALR-6 overexpressing Nalm-6 cell lines, as measured by MTS. **d** Representative histogram of Nalm-6 overexpression lines, stained with propidium iodide, shows an increase in S phase cells and G2-M cells. **e**, **f** Increased cell proliferation in BALR-6 Isoform 1 (**e**) and Isoform 3 (**f**) overexpressing 70Z/3 cell lines, as measured by MTS. **g**, **h** Propidium iodide staining of 70Z/3 cells overexpressing BALR-6 Isoform 1 (**g**) and Isoform 3 (**h**), shows a consistent increase in G2-M cells, and a decrease in Sub-G0 cells. Representative histogram of figures (**g-h**) confirmed the increase in cells in the G2-M when compared to the empty vector, shown to the right. ISO1, Isoform 1; ISO2, Isoform 2; ISO3, Isoform 3. Evaluations were made using a two-tailed *T*-test, *p* < 0.05 (*); *p* < 0.005 (**); *p* < 0.0005 (***); *p* < 0.0001 (****)

To examine the effects of BALR-6 gain of function, we overexpressed the previously identified isoforms in the human B-ALL cell line Nalm-6, which has relatively low endogenous levels of the transcript (Figs. 2b and 3a). Gene transfer was conducted via a lentiviral expression system that has proven successful in our previous studies (Additional file 1: Figure S2E) [16]. Constitutive overexpression of BALR-6 Isoforms 2 and 3 led to a significant increase in proliferation as measured by MTS (Fig. 3c). In addition to an observed increase in overall growth rate, BALR-6 Isoforms 2 and 3 caused an increase in S phase cells and G2-M cells (Fig. 3d). Furthermore, AnnexinV staining showed significantly lower numbers of apoptotic cells under basal growth conditions in cell lines overexpressing any of the BALR-6 isoforms (Additional file 1: Figure S2G).

To overexpress BALR-6 in mouse cells, we constructed a set of MSCV-based bicistronic vectors (Fig. 3b, Additional file 1: Figure S2F). Successful overexpression of these constructs in murine pre B-ALL 70Z/3 cells led to a modest increase in proliferation (Fig. 3e and f). Cell cycle analysis of these lines showed an increase of S phase cells, G2-M cells (in Isoform 3 overexpressing lines) and a reduction in Sub-G0 cells, similar to the effects in Nalm-6 cells (Fig. 3g and h). Analysis by AnnexinV staining confirmed the lower number of apoptotic cells in Isoform 3 expressing cell lines (Additional file 1: Figure S2H). Moreover, these 70Z/3 Isoform 3 overexpression lines were less vulnerable to prednisolone-induced apoptosis (Additional file 1: Figure S2I). Conversely, siRNA-transduced RS4;11 cells were more prone to prednisolone-induced apoptosis (Additional file 1: Figure S2I). Therefore, knockdown and overexpression of BALR-6 had opposing phenotypes in B-ALL cell lines, and gain-of-function phenotypes were conserved in both human and mouse cells.

### Enforced BALR-6 expression promotes expansion of hematopoietic progenitor populations in vivo

Since BALR-6 is highly expressed in B-ALL, we tested the effects of constitutive expression in an in vivo model [16]. 5-FU enriched bone marrow was transduced with retrovirus expressing the BALR-6 Isoform 3 and transplanted into lethally irradiated hosts (Fig. 3 and Additional file 1: Figure S2F, 2H). Mice were followed with peripheral bleeds for 16 weeks and then sacrificed for analysis. Peripheral white blood cell counts were not statistically different between the control and experimental groups. However, mice with enforced expression of BALR-6 showed a trend towards lower red blood cell counts, hematocrit and platelet counts (Additional file 1: Figure S3A). Flow cytometry revealed a lower percentage of CD11b + myeloid cells and a higher percentage of B220+ B-cells, but no difference in CD3ε + T-cell percentage in the eGFP+ population of experimental mice (Additional file 1: Figure S3B-C).

**Fig. 4:**
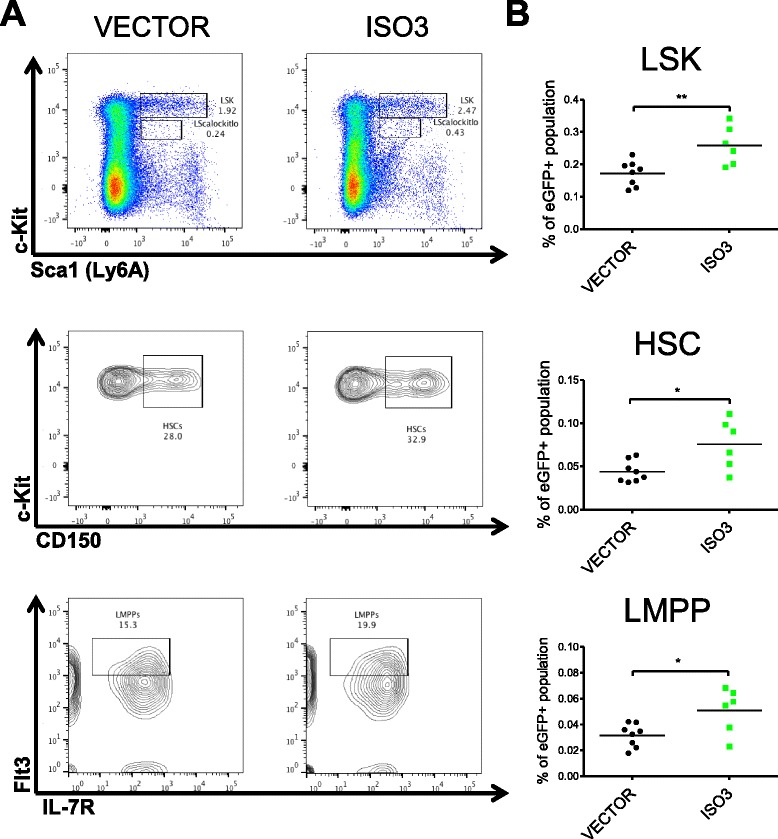
BALR-6 overexpression causes an increase in hematopoietic precursor cells in vivo. **a** Representative FACS plots of hematopoietic progenitor populations LSK, HSC and LMPP in bone marrow transfer mice. **b** Quantitation of progenitor populations showing a significant increase in experimental mice when compared to control. Number of mice used in this analysis: VECTOR, *n* = 8; ISO3, *n* = 6. ISO3, Isoform 3; HSC, hematopoietic stem cell; LMPP, lymphoid primed multipotent progenitor; LSK, lineage- Sca1+ c-Kit+. Evaluations made using a two-tailed *T*-test, *p* < 0.05 (*); *p* < 0.005 (**)

Mice were sacrificed following 4 months of reconstitution. Gross analysis showed no changes in the thymus, spleen, livers or kidneys. Microscopic inspection of hematoxylin and eosin – stained tissues did not reveal any differences (Additional file 1: Figure S3D). In the bone marrow, qRT-PCR confirmed successful overexpression of BALR-6 (Additional file 1: Figure S4A-B). Analysis by flow cytometry revealed an increase in precursor cell populations in the eGFP+ population of the experimental mice, when compared to the control group (Fig. 4a and b, Additional file 1: Figure S5C). After exclusion of differentiated cells in the bone marrow, we observed increased relative proportion of Lin-Sca1 + c-Kit + (LSK) cells, hematopoietic stem cells (HSCs) and lymphoid-primed multipotent progenitors (LMPPs) in mice overexpressing BALR-6 (Fig. 4a and b). An increase in the relative population of Lin-Sca1^lo^c-Kit^lo^ cells and a trend towards increased relative population of common lymphoid progenitors (CLPs) was also observed (Additional file 1: Figure S4C). The developmental pathway of B-cells in the bone marrow was investigated by the method of Hardy et al. [27]. Once again, trends towards higher relative proportions of these B-cell developmental stages were observed (fractions A-F, Additional file 1: Figure S4D). Taken together, these results suggest that BALR-6 overexpression leads to an enrichment of early developmental stage cells in murine bone marrow, indicating that its expression confers a survival advantage or increased proliferation for cells in these earlier stages.

### BALR-6 regulates expression of genes involved in multiple biological processes

**Fig. 5:**
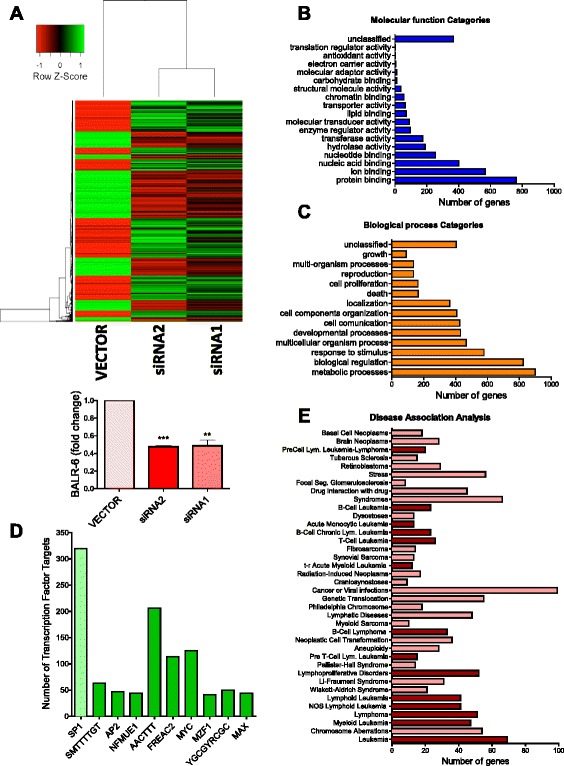
BALR-6 knockdown leads to global differential expression of genes. **a** Unsupervised hierarchical gene clustering of differentially expressed genes upon BALR-6 siRNA mediated knockdown in RS4;11 cells (PPDE ≥ 95 %, fold change ≥ 1.5). qRT-PCR confirmation of BALR-6 knockdown shown below. Normalized to ACTIN. **b**, **c** Bar graphs of GO Slim classification enrichment analysis of differentially expressed genes by molecular function (**b**), and biological processes (**c**). **d** Enrichment analysis of transcription factor targets. Top ten transcription factors with a *p*-value ≤ 0.0001 are shown. SP1 had the highest number if dysregulated targets, highlighted in light green. For unknown transcription factors, binding site sequence is shown. **e** Disease association analysis by GLAD4U, revealing enrichment of genes associated to various malignancies, in particular, hematological malignancies (*dark red*). Diseases with a *p*-value ≤ 0.05 are shown.; PPDE, posterior probability of differential expression. Evaluations were made using a two-tailed *T*-test, *p* < 0.005 (**); *p* < 0.0005 (***)

At the molecular level, several studies have demonstrated that many lncRNAs act as transcriptional regulators [5, 11, 23, 28, 29]. To explore whether or not BALR-6 regulates gene expression, RNA isolated from knockdown cell lines was analyzed by microarray [30, 31]. Upon siRNA mediated knockdown of BALR-6, 2499 probes showed differential expression. Of these, 1862 unambiguously mapped to 1608 unique Entrez Gene IDs. Unsupervised hierarchical clustering analysis identified differentially expressed genes in the siRNA-expressing cell lines (Fig. 5a).

Further data analysis was carried out using WebGESTALT [32, 33]. Gene Ontology (GO) slim classification of differentially expressed genes by molecular function was utilized to provide insight into the pathways in which BALR-6 is involved, with protein binding function category having the most dysregulated genes (Fig. 5b). A number of biological processes, as annotated in the GO database, were significantly dysregulated in BALR-6 knockdown cell lines, including cell death and cell proliferation (Fig. 5c). Disease associated enrichment analysis, which was inferred using GLAD4U, showed an enrichment of genes known to be dysregulated in various disease states (Fig. 5e). Of the 38 significantly associated disease states, 14 were of leukemic origin. Transcription factor enrichment analysis showed a significant enrichment of genes that are predicted targets of SP1, among other transcription factors (Fig. 5d). Taken together, these data revealed the biological importance of BALR-6. A detailed description of the microarray analyses can be found in the methods.

### SP1 transcriptome is modulated by BALR-6

**Fig. 6:**
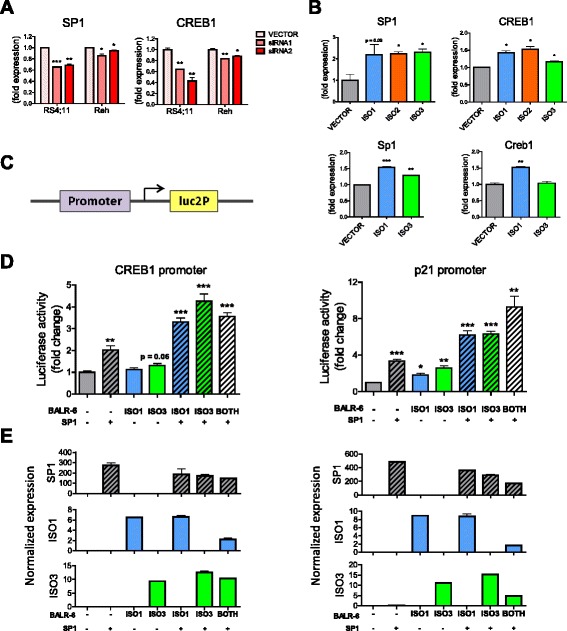
SP1 transcriptome is modulated by BALR-6. **a** Confirmation of SP1 and CREB1 expression in RS4;11 microarray samples, as well as Reh knockdown cell lines. Normalized to ACTIN. (**b**) SP1 and CREB1 transcript level increase correlates with overexpression of BALR-6 in Nalm-6 cells (*top*) and 70Z/3 cells (*bottom*). Quantitation by qRT-PCR, normalized to ACTIN (Nalm-6 cells) or L32 (70Z/3 cells). **c** Schematic depicting location of cloned promoter sequences in the pGL4.11 vector system for luciferase assays. **d** Transcriptional activity at CREB1 (*left*) and p21 (*right*) promoter regions upon SP1 and/or BALR-6 overexpression, as measured by luciferase activity. **e** Quantitation of overexpression in luciferase assays (as seen in (**d**)) by qRT-PCR of respective transcripts, normalized to ACTIN. Evaluations were made using a two-tailed *T*-test, *p* < 0.05 (*); *p* < 0.005 (**); *p* < 0.0005 (***). luc2p, synthetic firefly luciferase

As indicated by the transcription factor enrichment analysis, we confirmed that the expression SP1 and CREB1, a target and interactor of SP1, were dysregulated upon BALR-6 knockdown (Fig. 6a). The strongest phenotype was seen in the siRNA2-mediated knockdown, which also showed the strongest cellular phenotypes in the majority of pre B-ALL cell lines (Figs. 2d, i and 6a, Additional file 1: Figure S2B-D and Figure S5A-B). Conversely, increased levels of SP1 and CREB1 correlated with overexpression of BALR-6 isoforms in both human and murine cell lines (Nalm-6 and 70Z/3) (Fig. 6b).

To confirm our findings, a second microarray analysis was carried out with technical duplicates of RS4;11 cell lines transduced with empty vector or siRNA2. 2756 probes showed differential expression. Of these, 2280 unambiguously mapped to 2128 Entrez Gene IDs and were analyzed by hierarchical clustering (Additional file 1: Figure S6A). Enrichment analysis in WebGESTALT revealed similar GO slim classifications (Additional file 1: Figure S6B-C), and transcription factor target enrichment analysis confirmed the significant enrichment of SP1 targets seen previously (Fig. 5d, Additional file 1: Figure S6D). Additionally, enrichment of CREB1 targets was significant (Additional file 1: Figure S6D). Notably, leukemic diseases were the only ones significantly enriched in the disease association analysis (Additional file 1: Figure S6E). Together, these findings indicated a consistent change in the transcriptome, particularly downstream of SP1, upon knockdown of BALR-6 in MLL rearranged B-ALL.

To further understand the relationship of BALR-6 and SP1, we examined promoter regions of known SP1 targets (CREB1 and p21) and cloned these sequences into the luciferase reporter vector, pGL4.11 (Fig. 6c). The CREB1 promoter contained 7 putative SP1 binding sites, while the p21 promoter contained 6 such sites (Additional file 1: Figure S5C-D). Luciferase reporter assays in HEK 293 T cells with constitutive expression of SP1, Isoform 1, Isoform 3 or a combination of these vectors, revealed increased luciferase activity in both promoters (Fig. 6d and e). Notably, when SP1 and BALR-6 were co-overexpressed, we noted a strong increase in transcriptional activity with both the CREB1 and p21 promoter.

## Discussion

The discovery of lncRNAs has revolutionized how we think about gene expression. The genomic organization of many lncRNAs is indeed complex. Some are found in regions overlapping with protein coding genes, while others that are exclusively intergenic [2, 4]. Some lncRNAs contain microRNAs within either their exonic or intronic sequence [34, 35]. Here, we have characterized several isoforms of a lncRNA that is overexpressed in leukemia and shows dynamic expression in hematopoietic development [16]. Expressed from a locus adjacent to genes important in lymphocyte development, BALR-6 itself is dynamically regulated during human B-cell development [36–38]. Our work significantly adds to the known repertoire of RNA molecules that are expressed from this locus, and several of these appear to be functional within a cellular context.

In this manuscript, we describe the cellular function of a second lncRNA that was discovered as being overexpressed in MLL-translocated B-ALL. In some ways, BALR-6 shows some similarities with the other lncRNA we studied, BALR-2 [16]. Indeed, knockdown of both lncRNAs led to decreased cell growth and increased apoptosis, and overexpression led to increased growth and a partial resistance to prednisolone treatment. These findings are not altogether surprising given that these lncRNAs may be contributing to the poor clinical behavior of an aggressive cytogenetic subtype of B-ALL [17]. However, there are important differences between these lncRNAs—the genomic locus for BALR-6 is more complex, there are multiple isoforms and no comparable murine transcript is described in publically available databases. Nonetheless, we have obtained fragments of a low-expression transcript from murine hematopoietic cell lines that encoded portions homologous to human BALR-6. Further characterization of the murine transcripts will be the goal of future studies.

Significantly, our study is amongst the few characterizations of lncRNA dysregulation in the hematopoietic system [16, 39–41]. LncRNAs have been ascribed functions in lymphopoiesis, myelopoiesis and erythropoiesis [42–45]. Additionally, their differential expression has been described in peripheral T-cell subsets [46]. Here, we discovered the effect of BALR-6 overexpression on early hematopoietic progenitors in the marrow, including LSK cells, HSCs and LMPPs. Constitutive expression of BALR-6 isoforms led to increased survival or proliferation of normally transient bone marrow progenitor cells. Furthermore, Hardy fractions showed a trend towards being increased when compared to control, particularly those that developmentally precede the large pre B-cell stage (fraction C’, early pre-B). The relative percentages of more mature B-lineage cells downstream of these developmental stages are largely normal. Despite increased proportions of early progenitor cells, passage through a checkpoint (such as the pre-BCR checkpoint) may reduce cell numbers back to baseline. This suggests that the function of BALR-6 in vivo may be in directing differentiation and adequate lymphoid cell development. The upregulation of this lncRNA causes a survival or proliferative advantage, a hallmark of leukemogenesis. Coupling BALR-6 overexpression with an appropriate oncogenic co-stimulus may lead to full-blown leukemogenesis or enhancement thereof, and this is currently an active area of investigation in the laboratory.

In line with a function in promoting the survival of early hematopoietic progenitors, BALR-6 clearly affects proliferation in cell line experiments. Upon siRNA mediated knockdown, we saw reduced cell proliferation and increased cell death. We observed the opposite effect when we constitutively expressed BALR-6 in human and murine B-ALL cell lines. Moreover, similar mechanisms may be operant in B-ALL with MLL translocations, and loss-of-function experiments in primary patient samples and mouse models of MLL-driven leukemia are areas for further investigation.

Given prior reports of lncRNAs serving to regulate transcriptional complexes, our finding that BALR-6 knockdown causes changes in the SP1 transcriptome is compelling. SP1 is a transcriptional regulator that is associated with dysregulated cell cycle arrest in multiple myeloma [47–49]. CREB1 is a well-known proto-oncogene that promotes cellular proliferation in hematopoietic cells [50, 51]. Here we demonstrate that SP1-mediated transcription at the CREB1 and p21 promoters are positively regulated by BALR-6, providing a putative mechanism for our observations of BALR-6′s role in B-ALL.

## Conclusions

In this study, we demonstrate that the MLL-AF4-dysregulated lncRNA, BALR-6, plays a role in cell survival and regulates hematopoietic progenitors. At the molecular level, BALR-6 regulates the transcriptome of B-ALL cell lines, likely through regulating SP1-mediated transcription. In summary, our study has several novel and unique findings that help uncover a role for a poorly understood class of molecules in a pathogenetic process. This will undoubtedly have impacts on our understanding of molecular biology within cancer cells.

## Methods

### Cloning and cell culture

mmu-miR-155 formatted siRNAs were cloned into BamHI and XhoI sites in the pHAGE2-CMV-ZsGreen-WPRE vector using the strategy that we have previously described to generate knockdown vectors [16, 25, 26, 52]. Using the sequence information from 5′ and 3′ RACE products we cloned full length transcripts into an MSCV-based bicistronic viral vector between the BamHI and XhoI sites, as described previously and into a pHAGE6-UBC-ZsGreen-CMV-LNC (P6UZCL) variant of the third generation lentiviral vector system, between the NotI and BamHI sites [16, 52]. Primer sequences used are listed in Additional file 2: Table S1 or mentioned previously [16]. RS4;11 and MV(411), (MLL-AF4-translocated; ATCC CRL-1873 and CRL-9591), Reh (TEL-AML1-translocated; CRL-8286), 697 (E2A-PBX1-translocated), Nalm-6, 70Z/3 (ATCC TIB-158) murine pre B-cell leukemic cell line, and the HEK 293 T cell line (ATCC CRL-11268) were grown in their corresponding media at 37 °C in a 5 % CO_2_ incubator as previously described [16, 53].

### Rapid amplification of cDNA ends (RACE)

To determine the 5′ and 3′ transcript ends of the lncRNAs, we performed RACE using First Choice RLM-RACE kit (Ambion). Using the sequence information from 5′ and 3′ RACE products, we cloned full length transcripts into P6UZCL, and into the MSCV viral vector. Primer sequences used and isoform sequences obtained are listed in Additional file 2: Table S1.

### Transduction and sorting of cell lines

Lentiviruses and MSCV-based retroviruses were produced to generate knockdown constructs as previously described [16, 25, 26, 52]. In brief, 5.0 × 10^5^ cells were spin-infected at 30 °C for 90 min in the presence polybrene (4 μg/mL). Transduced cell lines were sorted for high green expression using a BD FACSAriaII cell sorter, and analysis was performed using BD FACSDiva software.

### Biological assays

For pharmaco-induced assays, cells were cultured at a concentration of 1.0 × 10^6^ cells per mL and treated for 36 h. I-BET151 was dissolved in dimethyl sulfoxide to desired concentrations. After treatment, cells were harvested for RNA extraction. For MTS proliferation assays, cells were cultured for at least 5 days before plating. Cells were plated at a density of 2500 cells per 100 μl of media in each well of a 96 well plate. Reagents were added according to the manufacturer’s instructions (Promega CellTiter 96 Aqueous Non-Radioactive Cell Proliferation Assay kit) and cells were incubated at 37 °C, 5 % CO_2_ for 4 h before absorbance was measured at 490 nm. For apoptosis assays, cells were plated at 5.0 × 10^5^ cells/mL for 24 h with or without prednisolone treatment. Prednisolone (TCI America) was dissolved in dimethyl sulfoxide to desired concentrations. Cells were harvested after 24 h and stained with APC-tagged AnnexinV. For cell cycle analysis, cells were synchronized by serum starvation for 12 h (human cell lines) or 4 h (murine cell lines) then plated at 5.0 × 10^5^ cells/mL and incubated at 37 °C, 5 % CO_2_ for 24 h. Cells were harvested, fixed with EtOH and then stained with propidium iodide. AnnexinV stained and PI stained samples were analyzed using a BD FACS HTLSRII flow cytometer and further analysis was performed using FlowJo.

### Luciferase assays

Promoter sequences for CREB1 and p21 were cloned upstream of synthetic firefly luciferase (luc2p) in the pGL4.11 vector. Renilla luciferase is expressed in the pGL4.75 vector downstream of the PGK promoter. HEK 293 T cells were transfected with the pGL4.75 and pGL4.11 containing reporter vectors at a 1:20 ratio (5 ng:100 ng), along with a combination of MSCV vector (empty, Isoform-1 or Isoform-3) and pCMV3 (empty or SP1-HA, Sino Biological Inc.) vector at a 1:1 ratio (200 ng:200 ng). For the last condition SP1, Isoform1 and Isoform 3 were transfected together at a ratio of 2:1:1 (200 ng:100 ng:100 ng). Co-transfections were performed with BioT (Bioland Scientific LLC) in 24 well plates as per the manufacturer’s instructions. Cells were lysed after 32 h and supernatant lysate was collected as per manufacturer’s instructions (Promega). The dual luciferase assay kit (Promega) was used as substrates for Renilla and firefly luciferase activity. Luminescence was measured on a Glomax-Multi Jr (Promega). The ratio of firefly to Renilla luciferase activity was calculated for all samples. The luminescence for the sample co-transfected with MSCV empty vector and pCMV3 empty vector, was used as a normalization control.

### qRT-PCR and PCR

RNA from cell lines was reverse transcribed using qScript (Quantas Biosciences). Real Time quantitative PCR was performed with the StepOnePlus Real-Time PCR System (Applied Biosystems) using PerfeCTa SYBR Green FastMix reagent (Quantas Biosciences). cDNA from mice samples was amplified using KOD Master Mix (EMD Millipore) and ran on a 1.2 % agarose gel stained with ethidium bromide. Primer sequences used are listed in Additional file 2: Table S1.

### Northern blot

Total RNA was separated on a 1.2 % (w/v) formaldehyde agarose gel and then blotted onto Hybond N+ nylon membranes (Amersham Biosciences) by semi-dry transfer (Bio-Rad,

Trans-Blot SD Semi-Dry Transfer Cell). DNA probes were ordered from Integrated DNA Technologies (IDT, San Diego, CA) with digoxigenin incorporated at 3′end. For ACTIN we used the RNA probe provided in the DIG Northern Starter Kit (Roche). Membranes were hybridized overnight using ULTRAhyb-Oligo Buffer (Ambion) at 37 or 42 °C with probes. Visualization was done by X-Ray film using CDP-Star reagents (Roche). X-Ray film was scanned and saved as jpeg files. Brightness and contrast was increased by 20 % for ease of visualization.

### Data sources

Human genome assembly GRCh37/hg19 and the mouse genome assembly GRCm38/mm10 were used. Methylation patterns for the four cell lines were obtained from Chip-Seq data available in the UCSC genome browser generated by the Broad/ENCODE group [18–20]. Peak viewing range set from 1–50 for H3K4m3 modifications, and 1–15 for H3K36m3 modifications. Alternative splice form information was obtained from the Swiss Institute of Bioinformatics, via UCSC Genome Browser [21]. Genome alignments of RefSeq transcripts from human, mouse and other vertebrates, GenBank mRNAs and ESTs, as well as PhastCons scores were obtained from the UCSC Genome Browser [22].

### Microarray data analysis

Microarray data was generated from samples of 3 different transduced RS4;11 cell lines with siRNAs against BALR-6, or the control empty vector. Samples were hybridized at the UCLA Clinical Microarray Core facility using Affymetrix HG-U133_Plus_2 microarray. The Affymetrix raw data files (.cel files) were loaded into the R program for quality control analysis. Additionally, raw hybridization intensities were normalized using the MAS5 method with the affy package in R. Normalized values were sorted by detection *p*-value ≤ 0.05. Differential expression analysis was performed using unpaired Bayesian comparison model (CyberT Website) [30, 31]. Data was then sorted for genes with a posterior probability of deferential expression (PPDE) ≥ 95 % and a fold change ≥ 1.5. Analysis of differentially expressed genes was carried out using the WEB-based GEne SeT AnaLysis Toolkit (WebGESTALT, http://bioinfo.vanderbilt.edu/webgestalt/) [32, 33]. This online tool uses information from different public data sources for enrichment analysis, including the Gene Ontology data base, and GLAD4U. A second (validation) microarray was carried out, as described above, with technical duplicates for RS4;11 cell lines transduced with siRNA2 or the empty vector. For differential analysis the raw data files were loaded into the R environment and analyzed using the R library of Linear Models for Microarray Data (LIMMA). Pairwise comparison and eBayes fit was carried out. Data was then sorted for genes with a *p*-value ≤ 0.05. Further analysis was done as described above, using WebGESTALT.

### Mice and bone marrow transplantation

Mice were housed under pathogen free conditions at the University of California, Los Angeles (UCLA). Donor mice were injected intraperitoneally with 200 mg/kg of 5-fluorouracil. After 5 days the mice were sacrificed. The bone marrow was collected under sterile conditions and plated in media enriched with IL-3, IL-6 and mSCF (Gibco). 24 h after plating, the bone marrow was spin infected twice, at 30 °C for 90 min in the presence polybrene (4 μg/mL), with retroviruses expressing the empty MSCV vector or BALR-6 Isoform 3. Recipient mice were lethally irradiated and injected with donor bone marrow 6 h after irradiation. 8 mice were used per group. One mouse in the ISO3 group died due to engraftment failure after 2 weeks post injection. These mice were bled at 8, 12 and 16 weeks post bone marrow injection. At 16 weeks the mice were sacrificed for full analysis. For statistical analysis, one mouse was excluded due to low eGFP expression. This experiment was repeated, and had similar results. All animal studies were approved by the UCLA Animal Research Committee (ARC).

### Flow cytometry of samples

At 16 weeks post bone marrow transplant, blood, bone marrow, thymus and spleen were collected from the mice under sterile conditions [53]. Single cell suspensions were lysed in red blood cell lysis buffer. Fluorochrome conjugated antibodies were used for staining (antibodies were obtained from eBiosciences, and Biolegend). Cells were stained with surface marker antibodies for 30 min at 4 °C, washed twice with 1X PBS, and finally fixed with 1 % PFA. Flow cytometry was performed at the UCLA Jonsson Comprehensive Cancer Center (JCCC) and at the BROAD Stem Cell Research Flow Core. Analysis was performed using FlowJo software. The lists of antibodies used and gating schematics are provided in Additional file 2: Table S2. Normal adult human bone marrow was obtained commercially from healthy adults (All Cells, Inc.) as previously described [51]. CD34 enrichment from human bone marrow was performed using the magnetic activated cell sorting (MACS) system (Miltenyi Biotec, San Diego, CA) prior to isolation of CD34+ subsets by flow cytometry. Bone marrow CD34 selected cells were incubated with a cocktail of antibodies as well as FITC-labeled lineage depletion antibodies (Additional file 2: Table S3). CD19 was not included in the lineage depletion cocktail used for sorting the progenitor B population. The immunophenotypic definitions used to isolate progenitors from human bone marrow CD34 selected cells are described in Additional file 2: Table S3. All populations were purified using fluorescence-activated cell sorting on a FACSAria (355, 405, 488, 561 and 633 nm lasers) (BD Immunocytometry Systems).

## Additional files

Additional file 1: Figure S1. BALR-6 locus encodes numerous alternative splice forms. **Figure S2.** Knockdown and overexpression of full length BALR-6 isoforms in mammalian cell lines. **Figure S3.** Constitutive expression of BALR-6 in mice periphery. **Figure S4.** Elevated levels of immature B cell populations in mice with BALR-6 overexpression. **Figure S5.** SP1 targets in siRNA mediated knockdown cell lines. **Figure S6.** Confirmation of global differential expression findings seen in initial microarray. (PDF 1.19 mb) Additional file 2: Tables S1. Primers and RACE sequences for BALR-6. **Table S2.** Antibodies used for bone marrow transplant flow cytometry analysis, and population gating schematics. **Tables S3.** Antibodies used for CD34 enrichment of human bone marrow flow cytometry analysis, and population gating schematics. (PDF 125 kb)

## Abbreviations

BALR: B-ALL associated long RNA
chr: chromosome
CLP: common lymphoid progenitor
CMV: cytomegalovirus promoter
DMSO: dimethyl sulfoxide
eGFP: enhanced green fluorescent protein
HSC: hematopoietic stem cell
ISO1: Isoform 1
ISO2: Isoform 2
ISO3: Isoform 3
LMPP: lymphoid primed multipotent progenitor
LSK: lineage- Sca1^+^ c-Kit^+^
LTR: long terminal repeats
ORF: open reading frame
PFA: paraformaldehyde
PGK: phosphoglycerate kinase promoter
PPDE: posterior probability of differential expression luc2p, synthetic firefly luciferase
pre-B: precursor B
pro-B: progenitor B
UBC: ubiquitin C promoter
ZsGreen: Zoanthus green fluorescent protein
