# Supplementary material for: BALR-6 regulates cell growth and cell survival in B-lymphoblastic leukemia
Source: Mol Cancer. 2015 Dec 22;14:214. doi: 10.1186/s12943-015-0485-z (PMC4688921; doi:10.1186/s12943-015-0485-z)
Supplement: Additional file 1: Figure S1. — BALR-6 locus encodes numerous alternative splice forms. Figure S2. Knockdown and overexpression of full length BALR-6 isoforms in mammalian cell lines. Figure S3. Constitutive expression of BALR-6 in mice periphery. Figure S4. Elevated levels of immature B cell populations in mice with BALR-6 overexpression. Figure S5. SP1 targets in siRNA mediated knockdown cell lines. Figure S6. Confirmation of global differential expression findings seen in initial microarray. (PDF 1.19 mb) [file 12943_2015_485_MOESM1_ESM.pdf]

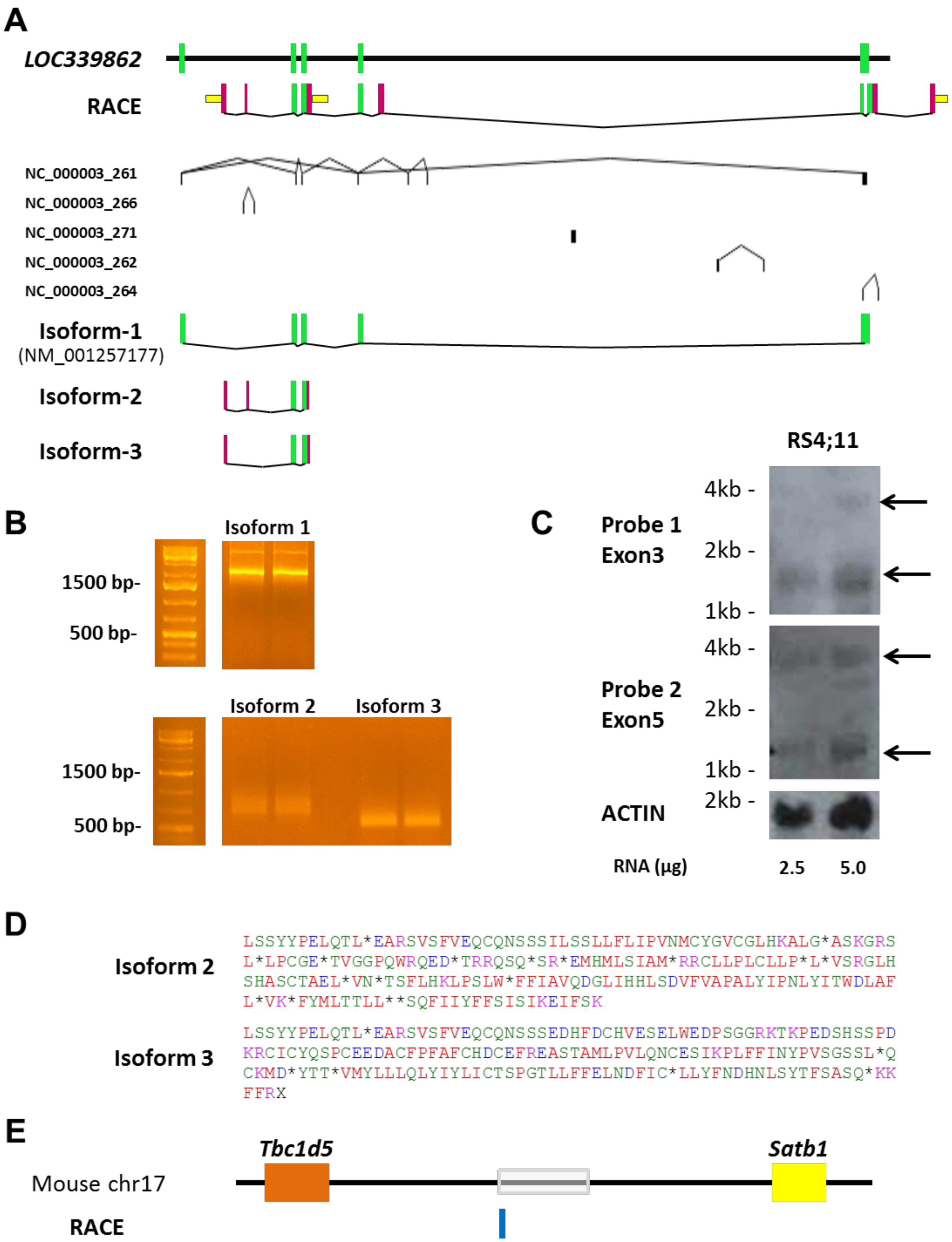

Supplemental Figure 1. BALR-6 locus encodes numerous alternative splice forms.
(**A**) Top: Diagram of RACE products obtained from *LOC339862*. 5' and 3' RACE primers are shown in yellow, with the newly discovered exons shown in magenta, as seen in Figure 1C. Known annotated exons are shown in green. Middle: Alternative splicing graph from the Swiss Institute of Bioinformatics of the predicted alternative splicing transcripts shown in the SIB Genes track. Blocks represent exons, lines indicate introns. Bottom: Schematic depiction of BALR-6 isoforms cloned from RACE sequences. Annotated exons in green, unannotated in magenta. (**B**) Gel confirmation of the isoforms cloned, including the annotated mRNA sequence (Isoform 1). (**C**) Northern blot of endogenous levels of two BALR-6 isoforms in RS4;11 cells. (**D**) EMBOSS analysis of the new isoforms confirmed lack of open reading frames, and lack of translation initiation sites. (**E**) Diagram of RACE products obtained from mouse cell lines with homology to BALR-6. chr, chromosome.

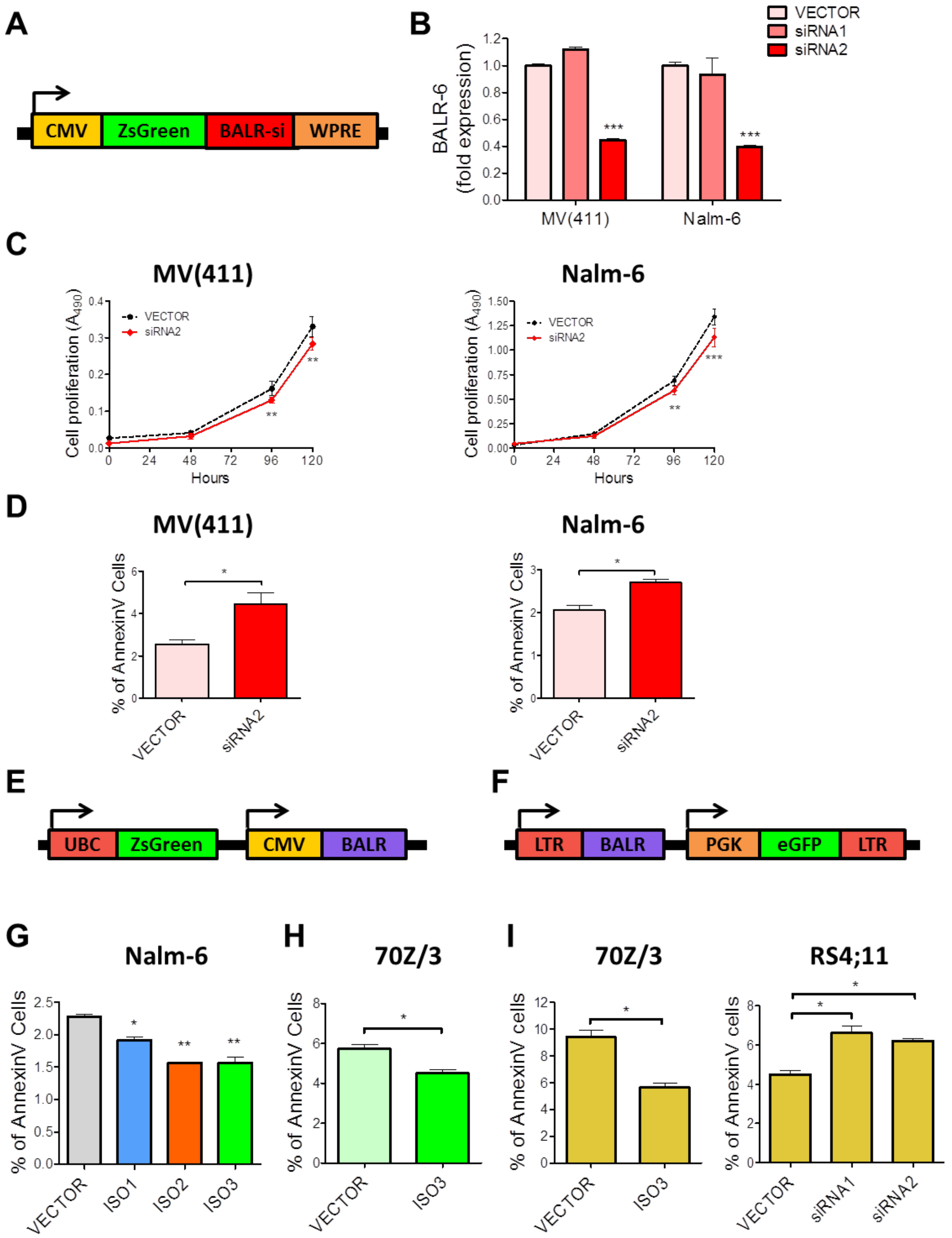

Supplemental Figure 2. Knockdown and overexpression of full length BALR-6 isoforms in mammalian cell lines.

(**A**) Schematic representation of mmu-miR-115 knockdown expression cassette. (**B**) Successful knockdown of BALR-6 using siRNA2 in MV(411), and Nalm-6 cells. (**C**) Decreased cell proliferation in transduced MV(411), and Nalm-6 lines as measured by MTS assay. (**D**) Increased apoptosis at basal levels in MV(411), and Nalm-6 stable lines as measured by AnnexinV staining. (**E-F**) Schematic representation of dual promoter phage (**E**) and MSCV (**F**) expression cassettes. (**G**) AnnexinV staining showed that Nalm-6 stably transduced with BALR-6 isoforms, had lower number of apoptotic cells at basal level. (**H**) 70Z/3 cells overexpressing BALR-6 Isoform 3 had fewer apoptotic cells at basal level, as analyzed by AnnexinV staining. (**I**) 70Z/3 cells stably transduced with BALR-6 Isoform 3, resulted in reduction of apoptosis upon treatment with 250 µg/mL prednisolone for 6 hours. The opposite effect was seen with RS4;11 cells with siRNA mediated knockdown of BALR-6 and treated with 250 µg/mL prednisolone for 24 hours. Evaluations were made using a two-tailed T-test, $p<0.05$ (*); $p<0.005$ (**); $p<0.0005$(***). UBC, ubiquitin C promoter; ZsGreen, Zoanthus green fluorescent protein; CMV, cytomegalovirus promoter; LTR, long terminal repeats; PGK, phosphoglycerate kinase promoter; eGFP, enhanced green fluorescent protein.

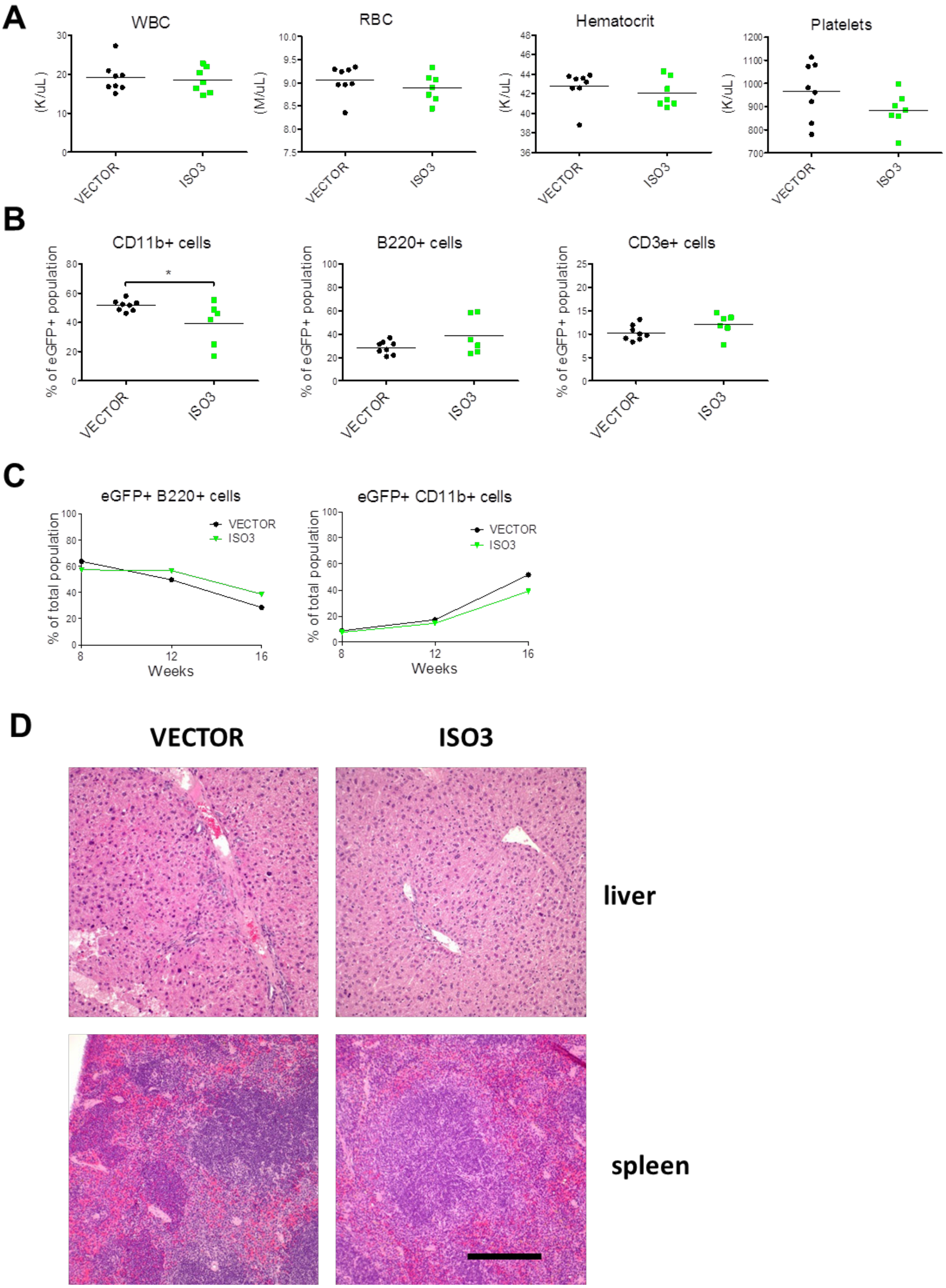

Supplemental Figure 3. Constitutive expression of BALR-6 in mice periphery.
(**A**) Peripheral white and red blood, hematocrit, and platelet cell counts. (**B**) Levels of B-cells (B220+), T-cells (CD3e+) and Myeloid cells (CD11b+) in the eGFP+ compartment of the peripheral blood at 16 weeks. (**C**) Average levels of eGFP+ B cells (B220+) and eGFP+ Myeloid (CD11b+) cells in the peripheral blood throughout the experiment. Number of mice used in this analysis: VECTOR, n=8; ISO3, n=6. (**D**) Hematoxylin and eosin stained liver and spleen samples from bone marrow transfer mice. Scale bar, 200 mm. ISO3, Isoform 3. Evaluations made using a two-tailed T-test, $p<0.05$ (*).

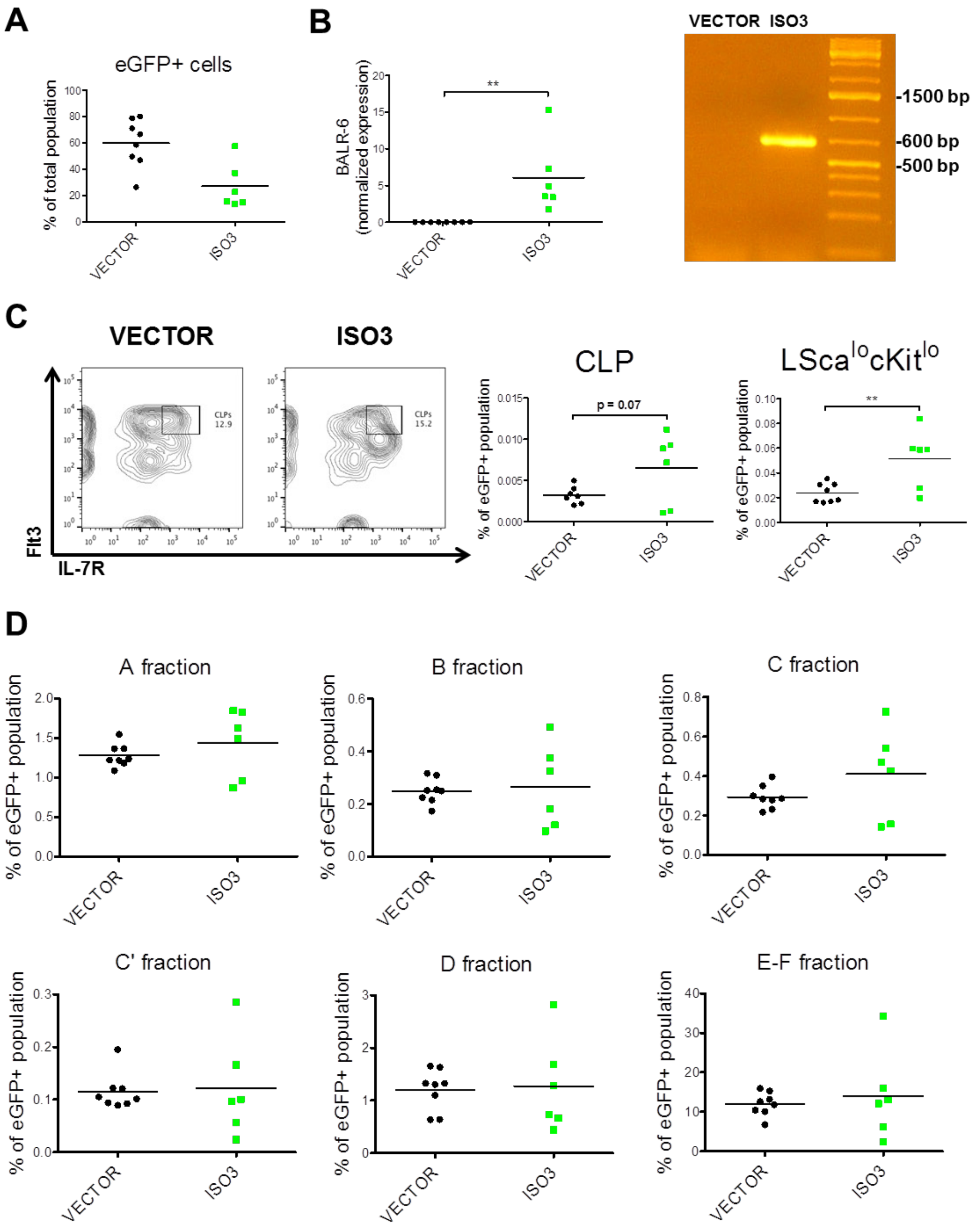

Supplemental Figure 4. Elevated levels of immature B cell populations in mice with BALR-6 overexpression.

(**A**) Levels of e+ cells in the bone marrow of experimental mice. (**B**) Expression levels of BALR-6 Isoform 3 in the experimental mice by qRT-PCR. Normalized to L32. Gel of cDNA PCR, obtained from bone marrow samples, confirming expression of full length transcript, shown to the right. (**C**) Representative FACS plots of CLP cells in the eGFP+ compartment of the experimental mice. Quantitation of the population gating and of LSca^{lo}c-Kit^{lo} cells, shown to the right. (**D**) Percentage of cells in the Hardy fractions from the eGFP+ compartment of experimental mice. Number of mice used in this analysis: VECTOR, n=8; ISO3, n=6. ISO3, Isoform 3. Evaluations made using a two-tailed T-test, p<0.005 (**).

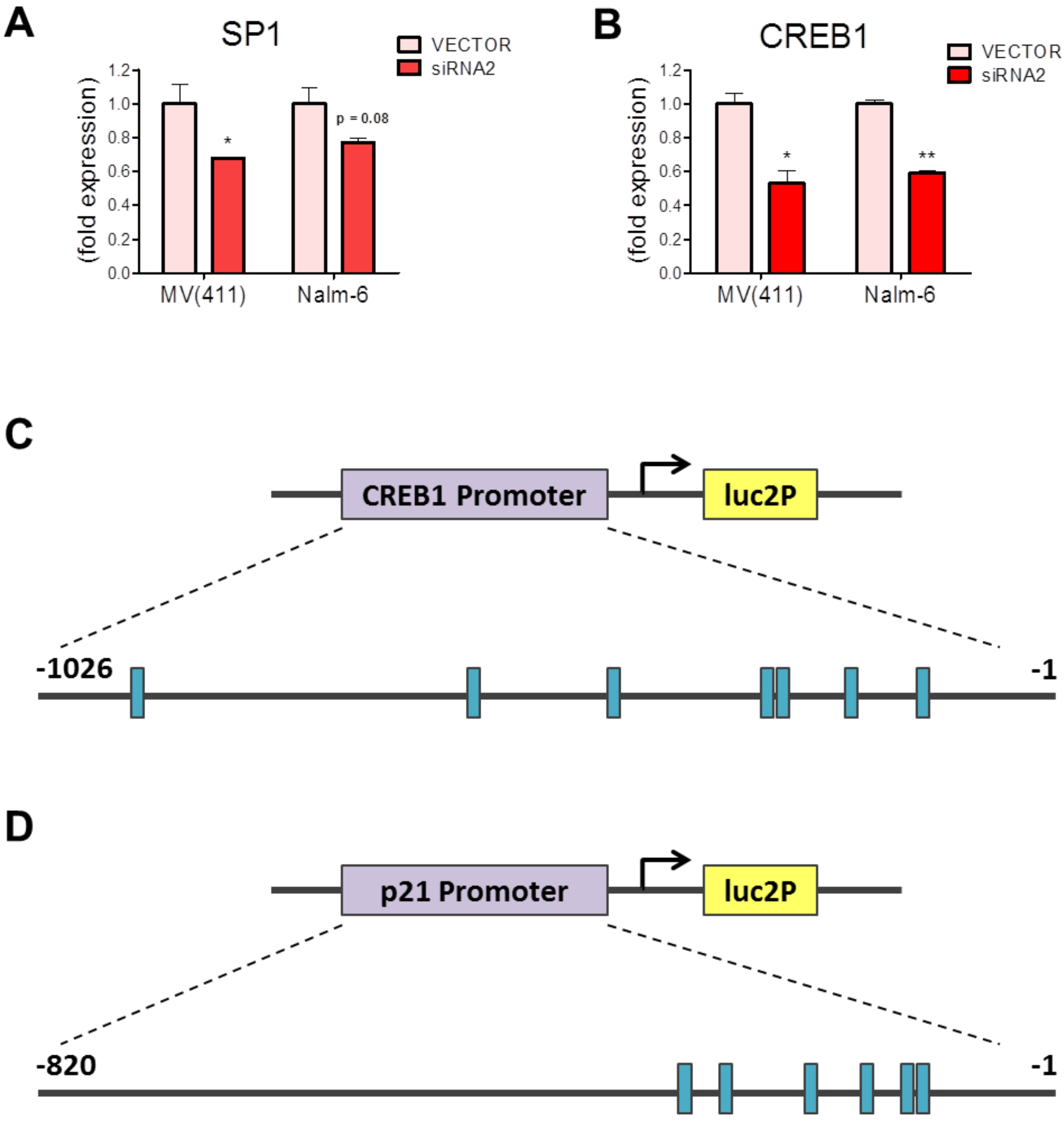

Supplemental Figure 5. SP1 targets in siRNA mediated knockdown cell lines.
(**A-B**) Transcript levels of SP1 (**A**) and CREB1 (**B**) in MV(411) and Nalm-6 knockdown cells. qRT-PCR quantitation of expression, normalized with ACTIN. Only expression levels upon siRNA2 mediated knockdown, which was successful, are shown. (**C-D**) Schematic representation of CREB1 (**C**) and p21 (**D**) promoter sequences cloned into the pGL4.11 luciferase expression vector. Promoter sequence distance shown in relation to the luc2p start codon. SP1 binding sites shown as blue boxes. luc2p, synthetic firefly luciferase.

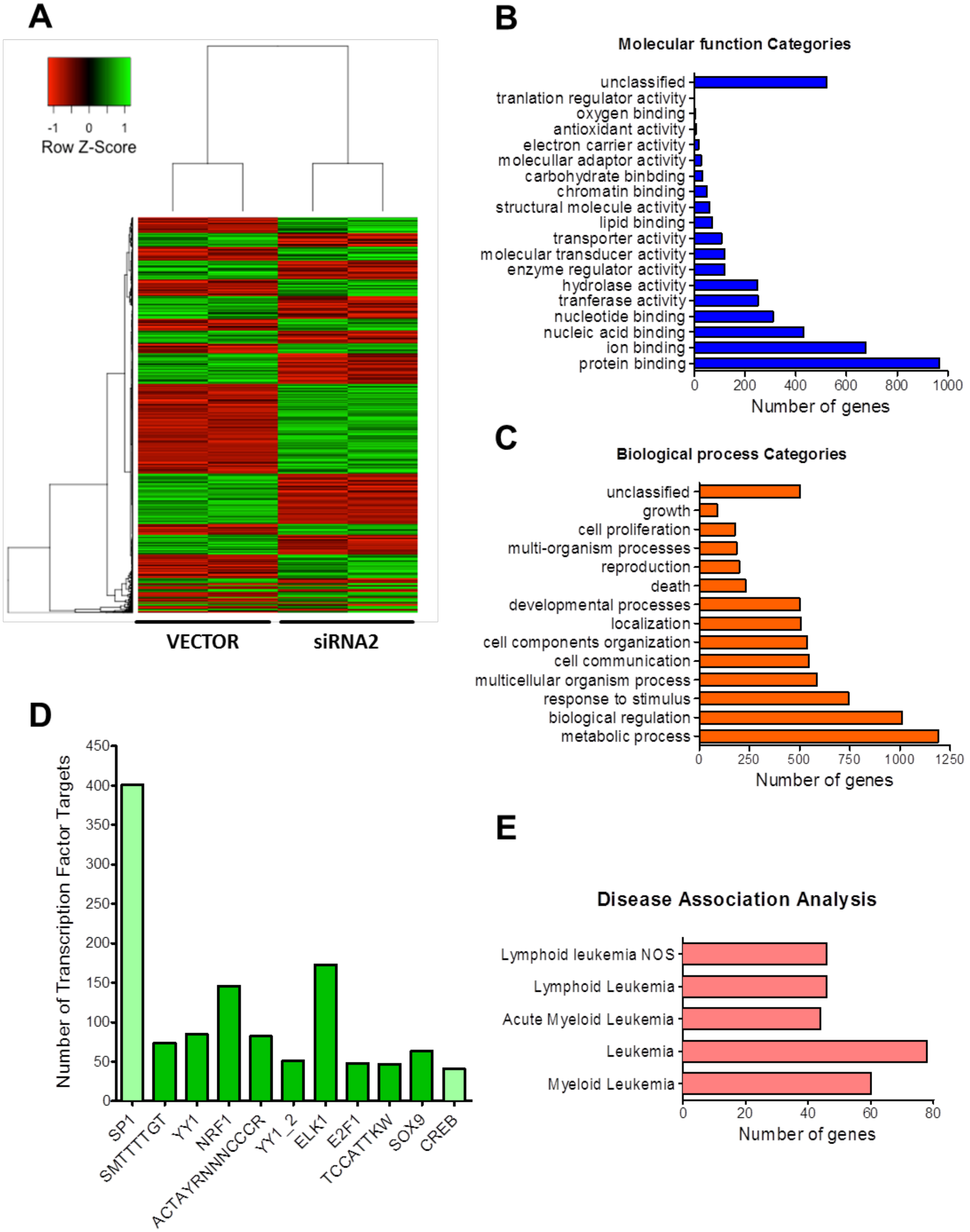

Supplemental Figure 6. Confirmation of global differential expression findings seen in initial microarray.

(**A**) Hierarchical gene clustering of differentially expressed genes in validation microarray upon siRNA2-mediated knockdown of BALR-6 in RS4;11 cells, p-value ≤ 0.05. Technical replicates of samples shown. (**B-C**) Bar graphs of GO Slim classification enrichment analysis of differentially expressed genes by molecular function (**B**) and biological processes (**C**) as analyzed by WebGESTALT. Proportions are highly similar to initial microarray. (**D**) Enrichment analysis of transcription factor targets. Top ten transcription factors with a p-value ≤ 0.02 are shown. In addition, CREB1 was shown as a significantly enriched for its targets with p-value = 0.04. For unknown transcription factors, transcription site sequence is shown. SP1 (shown in light green) had the most dysregulated targets. (**E**) Disease association analysis by GLAD4U, revealed a significant enrichment of genes solely associated to leukemic diseases, p-value ≤ 0.05.
