# Supplementary material for: BALR-6 regulates cell growth and cell survival in B-lymphoblastic leukemia
Source: Mol Cancer. 2015 Dec 22;14:214. doi: 10.1186/s12943-015-0485-z (PMC4688921; doi:10.1186/s12943-015-0485-z)
Supplement: Additional file 2: Tables S1. — Primers and RACE sequences for BALR-6. Table S2. Antibodies used for bone marrow transplant flow cytometry analysis, and population gating schematics. Tables S3. Antibodies used for CD34 enrichment of human bone marrow flow cytometry analysis, and population gating schematics. (PDF 125 kb) [file 12943_2015_485_MOESM2_ESM.pdf]

Supplemental Table 1: Primers and RACE sequences for BALR-6

**RT-qPCR primers**		
BALR-6 Set 1	FOW	5' CGTGTGCTGGGGAAGGCACTG 3'
	REV	5' CCAGGCTCAGAGCAACACAGGGA 3'
BALR-6 Set 2	FOW	5' GATCACTTTGATTGCCATGTGGA 3'
	REV	5' ATCTCTTATCTGGACTACGGTGAC 3'
ACTIN	FOW	5' CATGTACGTTGCTATCCAGGC 3'
	REV	5' CTCCTTAATGTCACGCACGAT 3'
SP1	FOW	5' TGGCAGCAGTACCAATGGC 3'
	REV	5' CCAGGTAGTCCTGTCAGAACTT 3'
CREB1	FOW	5' TTAACCATGACCAATGCAGCA 3'
	REV	5' TGGTATGTTTGTACGTCTCCAGA 3'
Sp1	FOW	5' AGGGTCCGAGTCAGTCAGG 3'
	REV	5' CTCGCTGCCATTGGTACTGTT 3'
Creb1	FOW	5' TGTAGTTTGACGCGGTGTGT 3'
	REV	5' GCTGGTTGTCTGCTCCAGAT 3'
L32 (mouse)	FOW	5' AAGCGAAACTGGCGGAAAC 3'
	REV	5' TAACCGATGTTGGGCATCAG 3'
Actin	FOW	5' GCTACAGCTTCACCACCACA 3'
	REV	5' GGGGTGTTGAAGGTCTCAAA 3'
**Cloning primers for P6UZCL**		
NotI site-1	FOW	5'ATGGGCTTAGCTGCGGCCGCTTTCTTCATACTATCCAGAGCTCCAAA3'
BamHI site-1	REV	5'ATGGCAATTATCGGATCCTTTTTTTTTTTCGAAAAAATTTCTTTTATTGAGATGCT3'
NotI site-2	FOW	5'ATGGCA ATTGCGGCCGCCCACGCGTCCGGGACTGAGCA 3'
BamHI site-2	REV	5'ATGGGCTGATGATCATTTTTTTTTGGTTCATAGAAAGTATTTTCTTCTAGAGTCTC3'
**Cloning primers for MSCV**		
HindIII_eGFP	FOW	5'ATGGGCTTAGCTAAGCTTATGGTGAGCAAGGGCGAGGAGC3'
DraIII_eGFP	REV	5'ATGGCAATTATCCACCTGGTGTTTACTTGTACAGCTCGTCCATGCCGA3'
BclI_BglII site	FOW	5' ATGGGCTGAGGATCTCCACGCGTCCGGGACTGAGCA 3'
XhoI site-1	REV	5'ATGGCAATTCTCGAGTTTTTTTTTGGTTCATAGAAAGTATTTTCTTCTAGAGTCTC3'
BamHI_BglII site	FOW	5'ATGGGCTTAGCTGGATCCTTTCTTCATACTATCCAGAGCTCCAAA3'
XhoI site-2	REV	5'ATGGCAATTATCCTCGAGTTTTTTTTTTTCGAAAAAATTTCTTTTATTGAGATGCT3'
**Sequencing primers for MSCV**		
Upstream_Puro	FOW	5' GCTGTTCTCCTCTTCCTCATCTCC 3'
Upstream_eGFP	FOW	5' CTTTATCCAGCCCTCACTCCTTCTCT 3'

**RACE primers**		
3-RACE_6	FOW	5' CCATGTGAAGAAGATGCTGGCTTC 3'
3-RACE_7	FOW	5' TAGGAAGCCAGAAGCGTCTCCTTT 3'
3-RACE_8	FOW	5' GGAGGCAGGAAGACTAAACCAGAA 3'
5-RACE_2	REV	5' CTCGCGAAACTCACAATCATGGCA 3'
5-RACE_4	REV	5' ATCTTCCATGTGCATGTGGCTGCA 3'
**Northern probes**		
BALR-6 probe 1		5'GGGCACAGAGTGTTGCATGCTCATTTCTGTTGATTTTTAATTAGCAGTAATTCATTT/3DiG_N/3'
BALR-6 probe 2		5'CTGGAAATCTAGGATCAGGACTAGCCTAAATTAGTAGATCTATGTGATAGTATATTGGTA/3DiG_N/3'
**mmu-miR-155 formatted siRNA oligos**		
siRNA1		5'GAAGGCTGTATGCTGGTGAACATACCACTTACCATTGTTTTGGCCACTGACTGACAATGGTAAGGTATGTTCACCAGGACACAAGGCCTG3'
siRNA2		5'GAAGGCTGTATGCTGGACTTCTGCACACCATGCCTGGTTTTGGCCACTGACTGACCAGGCATGGTGCAGAAGTCCAGGACACAAGGCCTG3'
**BALR-6 sequences**		
Isoform-2		5'TTTCTTCATACTATCCAGAGCTCCAAACTTTGTAGGAAGCCAGAAGCGTCTCCTTTGTTGAACAGTGCCAAAATAGCAGCTCTATCCTTTCCTCTCTCCTCTTTCTGATTCCAGTCAATATGTGTTATGGAGTCTGTGGTCTCCACAAGGCCTTGGGATAGGCATCCAAAGGAAGATCACTTTGATTGCCATGTGGAGAGTGAACTGTGGGAGGACCCCAGTGGAGGCAGGAAGACTAAACCAGAAGACAGTCACAGTAGTCCAGATAAGAGATGCATATGTTATCAATCGCCATGTGAAGAAGATGCTTGCTTCCCCTTTGCCTTCTGCCATGATTGTGAGTTTCGCGAGGCCTCCACAGCCATGCTTCCTGTACTGCAGAACTGTGAGTCAATTAAACCTCTTTTCTTCATAAATTACCCAGTCTCTGGTAGTTCTTTATAGCAGTGCAAGATGGACTAATACACCACCTAAGTGATGTATTTGTTGCTCCAGCTCTATATATACCTAATTTGTACATCACCTGGGACCTTGCTTTTCTTTGAGTTAAATGATTTTATATGTTAACTACTCTACTTTAATGATCACAATTTATCATATACTTTTTCAGCATCTCAATAAAAGAAATTTTTTCGAAA3'
Isoform-3		5'TTTCTTCATACTATCCAGAGCTCCAAACTTTGTAGGAAGCCAGAAGCGTCTCCTTTGTTGAACAGTGCCAAAATAGCAGCTCTGAAGATCACTTTGATTGCCATGTGGAGAGTGAACTGTGGGAGGACCCCAGTGGAGGCAGGAAGACTAAACCAGAAGACAGTCACAGTAGTCCAGATAAGAGATGCATATGTTATCAATCGCCATGTGAAGAAGATGCTTGCTTCCCCTTTGCCTTCTGCCATGATTGTGAGTTTCGCGAGGCCTCCACAGCCATGCTTCCTGTACTGCAGAACTGTGAGTCAATTAAACCTCTTTTCTTCATAAATTACCCAGTCTCTGGTAGTTCTTTATAGCAGTGCAAGATGGACTAATACACCACCTAAGTGATGTATTTGTTGCTCCAGCTCTATATATACCTAATTTGTACATCACCTGGGACCTTGCTTTTCTTTGAGTTAAATGATTTTATATGTTAACTACTCTACTTTAATGATCACAATTTATCATATACTTTTTCAGCATCTCAATAAAAGAAATTTTTTCGAAA3'

Supplemental Table 2: Antibodies used for bone marrow transplant flow cytometry analysis, and population gating schematics.

Marker	Fluorochrome
CD3e	PE
CD11b	PE-Cy7
B220	PerCP-Cy 5.5
CD117	APC-Cy7
Sca1	PerCP-Cy 5.5
CD135	APC
CD127	PE-Cy7
CD150	PE
IgM	PE
CD43	APC
CD24	PE-Cy7
Ly51	APC-Cy7
**For lineage negative staining**	
Biotin	CD3e, CD4, CD8, B220, NK1.1, Ter119, TCR beta, TCR gamma-delta
Streptavidin	eFluor 450 (pacific Blue)
**Population**	**Defined markers**
HSC	Lin- CD117 hi Sca1 hi CD150++
LMPP	Lin- CD117 hi Sca1 hi CD135+ CD127-
CLP	Lin- CD117 lo Sca1 lo CD135+ CD127+
A	B220+ IgM- CD43+ CD24- Ly51-
B	B220+ IgM- CD43+ CD24+ Ly51-
C	B220+ IgM- CD43+ CD24+ Ly51+
D	B220+ IgM- CD43-
E and F	B220+ IgM+

Antibodies were procured from eBiosciences (San Diego, CA) or Biolegend (San Diego, CA).

Supplemental Table 3: Antibodies used for CD34 enrichment of human bone marrow flow cytometry analysis, and population gating schematics.

**Marker**	**Fluorochrome**
CD34	APC-Cy7
CD38	APC
CD10	PE-Cy7
CD20	FITC
CD45RA	PerCP-Cy 5.5
IgM	PerCP-Cy 5.5
**For lineage depletion**	
FITC	CD3, CD14, CD15, CD19, CD56, and CD235a
**Population**	**Defined markers**
HSC	Lin- CD34+CD38-
CLP	Lin- CD34+CD10+CD45RA+
progenitor B	Lin- CD34+CD19+
precursor B	CD34-CD10+CD19+IgM-CD20-
Immature B	CD34-CD19+IgM+CD20+

Antibodies were procured from Becton Dickinson (BD, San Jose, CA) or Biolegend (San Diego, CA).
